# Career perspectives for young cardiologists in the Netherlands: a steady increase in temporary positions

**DOI:** 10.1007/s12471-022-01736-1

**Published:** 2022-11-28

**Authors:** V. M. M. Vorselaars, M. Minneboo, W. C. Meijers, A. C. van der Heijden, D. Haroun, V. J. M. Baggen, W. R. Berger, G. P. J. van Hout

**Affiliations:** 1 Department of Cardiology, St. Antonius Hospital, Nieuwegein, The Netherlands; 2 Department of Cardiology, Amsterdam University Medical Centres, location Academic Medical Centre, Amsterdam, The Netherlands; 3 Department of Cardiology, University Medical Centre Groningen, Groningen, The Netherlands; 4 Department of Cardiology, Leiden University Medical Centre, Leiden, The Netherlands; 5 Department of Cardiology, Medisch Spectrum Twente, Enschede, The Netherlands; 6 Department of Cardiology, Erasmus Medical Centre, Rotterdam, The Netherlands; 7 Department of Cardiology, Onze Lieve Vrouwe Gasthuis, Amsterdam, The Netherlands; 8 Department of Cardiology, University Medical Centre Utrecht, Utrecht, The Netherlands

**Keywords:** Career perspectives, Young cardiologists, Temporary contracts

## Abstract

In the Netherlands, concerns have been raised regarding the high unemployment rates and the lack of permanent positions for young medical specialists. In the current study, we present data on contemporary early career perspectives in the field of cardiology. We conducted a survey among 304 young cardiologists who completed their training between 2015 and 2020; the response rate was 91%. Our analysis revealed a low unemployment rate (0.3%). One, 3 and 5 years after registration, 81%, 41% and 18% of the respondents, respectively, had not gained a permanent position. Having conducted a fellowship significantly delayed the time to permanent position (hazard ratio (HR): 0.48; 95% confidence interval (CI): 0.34–0.67). For those who had conducted a fellowship, holding a PhD degree (HR: 1.95; 95% CI: 1.10–3.44), age (per year increase, HR: 0.90; 95% CI: 0.82–0.99) and training in an academic hospital (HR: 1.97; 95% CI: 1.10–3.52) were of significant influence on the likelihood of having a permanent position at 3 years of follow-up. These results showed a disturbing increase in time to permanent position compared with an earlier analysis. This trend justifies close monitoring of the labour market in the coming years. Solutions for this multifactorial problem in the field of cardiology and across the entire medical specialty spectrum should be explored.

**Supplementary Information:**

The online version of this article (10.1007/s12471-022-01736-1) contains supplementary material, which is available to authorized users.

## Introduction

Due to a sharp increase in the demand for cardiovascular healthcare, the number of cardiologists working in the Netherlands has almost doubled between 2000 and 2021, to approximately 1100 [1, 2]. To be able to balance the labour market for medical specialists in the long term, the Dutch government has started to regulate enrolment in medical specialty training programmes since 1999. The estimated number of cardiology trainees required is determined annually, based on a 50-parameter model, which includes data on demographic characteristics of cardiologists, possible changes in the healthcare system and the estimated increase in healthcare demand [3].

However, it is uncertain whether use of this model truly results in a balanced labour market for cardiologists. One of the factors not considered in the model is the limitation of financial growth for hospital care as permitted by the Dutch government from 2019 onwards (*het hoofdlijnenakkoord*), which could contribute to labour market disruption [4, 5]. The COVID-19 pandemic has also been of influence, although this factor has not (yet) been included in the previously mentioned model [6].

While labour market modelling focuses on matching supply and demand, it does not take into consideration the type of employment contract (temporary or permanent). However, this is of vital importance for long-term job satisfaction and motivation among young cardiologists. To assess the prospects for this group, The Junior Board (*De Juniorkamer*) of the Netherlands Society of Cardiology (*Nederlandse Vereniging voor Cardiologie*) has started to monitor the Dutch labour market more than a decade ago [7, 8]. Herein, we present the results from the most recent and largest registry so far.

## Methods

### Digital questionnaire

In September 2021, a digital questionnaire was sent to all cardiologists who completed their training between January 2015 and December 2020, unless they already had a permanent position at the time of our previous survey [8]. Data were anonymised, and participants were asked to provide informed consent. The questionnaire included questions on demographic characteristics, professional profile, current employment status and previous jobs. Participants were also asked whether they experienced any problems on the job market.

### Statistical analysis

Descriptive statistics were used to describe participants’ characteristics and their type of employment contract. Categorical variables are expressed as number (percentage) and continuous variables as mean ± standard deviation (SD) or median (interquartile range). We used Kaplan–Meier analyses to assess the time required to obtain a permanent position. Log-rank statistics were used to compare groups. A univariate Cox regression analysis was used to analyse the association between likelihood of having a permanent position and age, gender, holding a Doctor of Philosophy (PhD) degree and type of teaching hospital attended. All statistical analyses were performed using Stata software version 16.0 (Stata Corp LP, College Station, TX, USA) and GraphPad Prism version 9.1.0 (GraphPad Software Inc., La Jolla, CA, USA).

## Results

Of the 304 invited cardiologists, 278 (91%) provided information and were included in the analysis. Mean ± SD age was 38 ± 3 years and 178 (64%) of the participants were male. Detailed demographic characteristics are shown in Table S1 (see in Electronic Supplementary Material). One cardiologist (0.3%) was unemployed at the time of the survey, and 42 cardiologists (15%) reported unemployment at any time during their early career. The duration of unemployment ranged from 1 to 9 months (see Table S1 in Electronic Supplementary Material).

Most cardiologists (84%) started their career on a temporary contract, the majority of whom as a fellow (41%) or attending physician (*chef de clinique*) (41%) (see Figure S1a in Electronic Supplementary Material). At the time of the survey, 41% of the respondents were on a temporary contract and 59% on a permanent contract, across different practices (see Figure S1b–c in Electronic Supplementary Material).

**Fig. 1:**
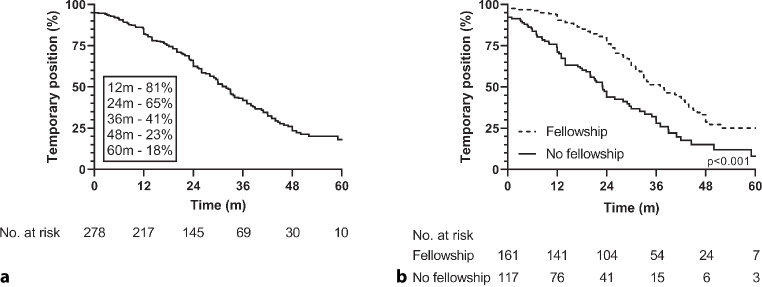
Temporary positions during follow-up of **a** all young cardiologists and **b** stratified by having conducted a fellowship or not

One year after entering the labour market. 81% of the participants had a temporary position (Fig. 1a). After 3 years of follow-up, 41% did not hold a permanent position; this percentage decreased to 18% after 5 years after follow-up.

Having conducted a fellowship (hazard ratio (HR): 0.48; 95% confidence interval (CI): 0.34–0.67) and age (per year increase, HR: 0.93; 95% CI: 0.87–0.99) reduced the likelihood of having a permanent position (Fig. 1b and Tab. 1). These results were consistent after 5 years of working experience (data not shown). Subanalyses for the group of young cardiologists who had conducted a fellowship revealed that holding a PhD degree (HR: 1.95; 95%: CI 1.10–3.44) and training in an academic hospital (HR: 1.97; 95% CI: 1.10–3.52) increased the likelihood of having a permanent position at 3 years of follow-up (Tab. 1). On the other hand, age (per year increase, HR: 0.90; 95% CI: 0.82–0.99) reduced the likelihood of having a permanent position. For cardiologists who had not conducted a fellowship, these parameters were not of significant influence (Tab. 1).

**Table 1:** Factors associated with likelihood of having a permanent position amongst young cardiologists after 3 years working experience^a^

Factor	HR (95% CI)^b^	*P*-value^c^
*Total cohort*		
Male sex	1.25 (0.88–1.78)	0.204
Academic teaching hospital	1.35 (0.93–1.95)	0.117
PhD	1.36 (0.95–1.95)	0.088
Conducted fellowship	**0.48 (0.34–0.67)**	**<0.001**
Age (per year increase)	**0.93 (0.87–0.99)**	** 0.018**
*Conducted fellowship*		
Male sex	0.88 (0.50–1.53)	0.648
Academic teaching hospital	**1.97 (1.10–3.52)**	** 0.022**
PhD	**1.95 (1.10–3.44)**	** 0.022**
Age (per year increase)	**0.90 (0.82–0.99)**	** 0.032**
*No fellowship*		
Male sex	1.23 (0.76–1.98)	0.399
Academic teaching hospital	1.04 (0.36–1.70)	0.887
PhD	1.35 (0.84–2.18)	0.216
Age (per year increase)	0.95 (0.88–1.03)	0.211

*PhD* Doctor of Philosophy degree

^a^Period of 3 years was based on cumulative incidence as calculated by Kaplan–Meier survival analyses

^b^Hazard ratio (*HR*) with 95% confidence interval (*CI*) was calculated using Cox regression analyses

^c^Significant *p*-values are shown in bold

Of all respondents, 77% experienced the current job market as problematic. The largest concern of young cardiologists was the lack of permanent positions.

## Discussion

The current study presented novel data on career perspectives for young cardiologists in the Netherlands. We demonstrated that the time to permanent position steadily increased over the last few years: at 3 years of follow-up, 41% of the young cardiologists did not have a permanent position compared with 30% (2011–2014) and 33% (2015–2018) in previous cohorts [7, 8]. Age and having conducted a fellowship significantly reduced the likelihood of having a permanent position, but no differences between genders were found. Although only one cardiologist was unemployed, it is important to emphasise that 15% of the young cardiologists reported temporary unemployment in the past.

At 5 years of follow-up, almost one out of five cardiologists did not have a permanent contract, which indicated this is a long-term issue for many. We also showed that 77% of the young cardiologists described the current job market as problematic, which is a substantial change compared with the 44% we observed earlier [8]. The unemployment rate in this 5‑year cohort was low and in line with previously published study results [7, 8]. This may suggest that at the time the survey was conducted, the enrolment rate of young cardiologists was balanced with the demand for cardiologists in the Dutch healthcare system. However, the low unemployment rate may be positively influenced by the exclusion of cardiologists who had entered the labour market in 2021 and selection bias (91% response rate), which are both limitations of the current analysis.

The gradual increase in time to permanent contract suggested an increasing problem in terms of career perspectives. This is a worrisome trend, which requires careful monitoring in the coming years. As we described in more detail in our previous paper, temporary employment is problematic for young cardiologists as it causes a high burden due to financial insecurity and geographical insecurity (i.e. because of job insecurity, young cardiologists need to travel far across the Netherlands to find a (temporary) job) during an important phase in their personal lives [8]. Temporary positions are accompanied by less autonomy and decreased control over their work environment [8]. These factors may consequently lead to a decline in job satisfaction and increased risk of burnout [9].

The problem of the increased number of temporary positions is not limited to cardiology and has been observed in many medical specialties in the Netherlands [10–14]. We believe this phenomenon reflects the uncertain financial situation of many Dutch hospitals and independent group practices. In the past 3 years, only very limited financial growth for medical specialty care was permitted [4, 5]. In this type of situation, temporary contracts may offer two main advantages over permanent positions: these contracts are overall less costly and refrain institutions from engaging in long-term (financial) relationships in an insecure labour market.

The short-term effect on an individual basis is that after a year in a temporary position, a cardiologist is forced to take up a different (temporary) position, while their former position is filled by another cardiologist, who also gets a temporary contract. The net workload in both institutions remains similar. The consequence of this ‘trading places’ phenomenon is not only the very limited career perspectives for a particular cardiologist but also the lack of continuous care (for example, for chronic outpatients) and transmural regional networks, which are important for modern patientcare. In these situations, there are financial and organisational hierarchies between the young cardiologist and their older counterparts with permanent positions, making these young cardiologists highly dependent and thereby vulnerable.

Solving this complex and multifactorial problem remains very challenging [15]. Taking away a financial incentive for offering a temporary rather than a permanent position could be a possible solution. However, this requires long-term reassurance of allocated financial budgets for group practices and hospitals. For the future of high-quality cardiology healthcare in the Netherlands, it is of great importance the government is convinced to invest in young cardiologists [16]. Another option is setting a cap on flexible employment contracts per institution to ensure the total number of temporary positions does not increase any further. There is, however, not a single organisation in the Netherlands that can force hospitals to limit this number. Therefore, these measures can only be based on consensual agreement among all stakeholders. Additionally, many other measures, such as increasing the number of part-time positions, mandatory retirement of cardiologists who have reached the statutory retirement age, gradual outflow of older cardiologists and job sharing between cardiologists could in theory have positive effects. In 2021, a special task force was initiated by the Netherlands Society of Cardiology to explore all possible solutions for this matter.

## Conclusion

Although no substantial unemployment among young cardiologists in the Netherlands was observed, the time to permanent contract was long and had increased over the last 10 years. This observation justifies close monitoring of the Dutch labour market for cardiologists in the coming years. Solutions for this problem in the field of cardiology as well as across the entire medical specialty spectrum should be continuously explored.

## Supplementary Information

**Table S1 **Baseline characteristics of young cardiologists

**Figure S1 **Employment of young cardiologists with regard to **a** first job, **b** type of position at time of survey, and **c** current job, divided into employed physician practice (*specialist in dienstverband*), attending physician (*chef de clinique*), fellow, independent group practice (*specialist in maatschap*), specialist employed by independent group practice (*specialist in dienst van maatschap*; *SPIDMA*) and other (e.g. cardiologist in private clinic)
