# Supplementary material for: Career perspectives for young cardiologists in the Netherlands: a steady increase in temporary positions
Source: Neth Heart J. 2022 Nov 28;31(5):177–80. doi: 10.1007/s12471-022-01736-1 (PMC9703432; doi:10.1007/s12471-022-01736-1)
Supplement: Supplementary file 1 — Table S1 Baseline characteristics of young cardiologists [file 12471_2022_1736_MOESM1_ESM.docx]

**Table S1** Baseline characteristics of young cardiologists

| Demographics | N=278 |
| --- | --- |
| Age | 38 ± 3 |
| Sex (male) | 178 (64.0%) |
| *Teaching hospital* |  |
| Academic | 149 (53.6%) |
| Non-academic | 129 (46.4%) |
| PhD (yes) | 167 (60.1%) |
| *Area of expertise during training* |  |
| Imaging | 94 (33.8%) |
| Clinical electrophysiology | 28 (10.1%) |
| Devices | 50 (18.0%) |
| Interventional cardiology | 63 (22.7%) |
| Adult congenital heart disease | 11 (4.0%) |
| Heart failure | 33 (11.9%) |
| General cardiology | 44 (15.8%) |
| Other | 20 (7.2%) |
| Fellowship (yes)* | 161 (57.9%) |
| Unemployment at time of survey | 1 (0.3%) |
| Endured unemployment | 42 (15.1%) |
| Months of unemployment | 3 [2-6] |
| Number of contracts per cardiologist | 2.4 ± 1.4 |

* This number represents cardiologists who are either currently enrolled in, or have already completed a fellowship. Continuous variables are presented as mean±standard deviation or median [interquartile range] and categorical variables as N (%). PhD = Doctor of Philosophy.
