# Supplementary figures and images for: Career perspectives for young cardiologists in the Netherlands: a steady increase in temporary positions
Source: Neth Heart J. 2022 Nov 28;31(5):177–80. doi: 10.1007/s12471-022-01736-1 (PMC9703432; doi:10.1007/s12471-022-01736-1)

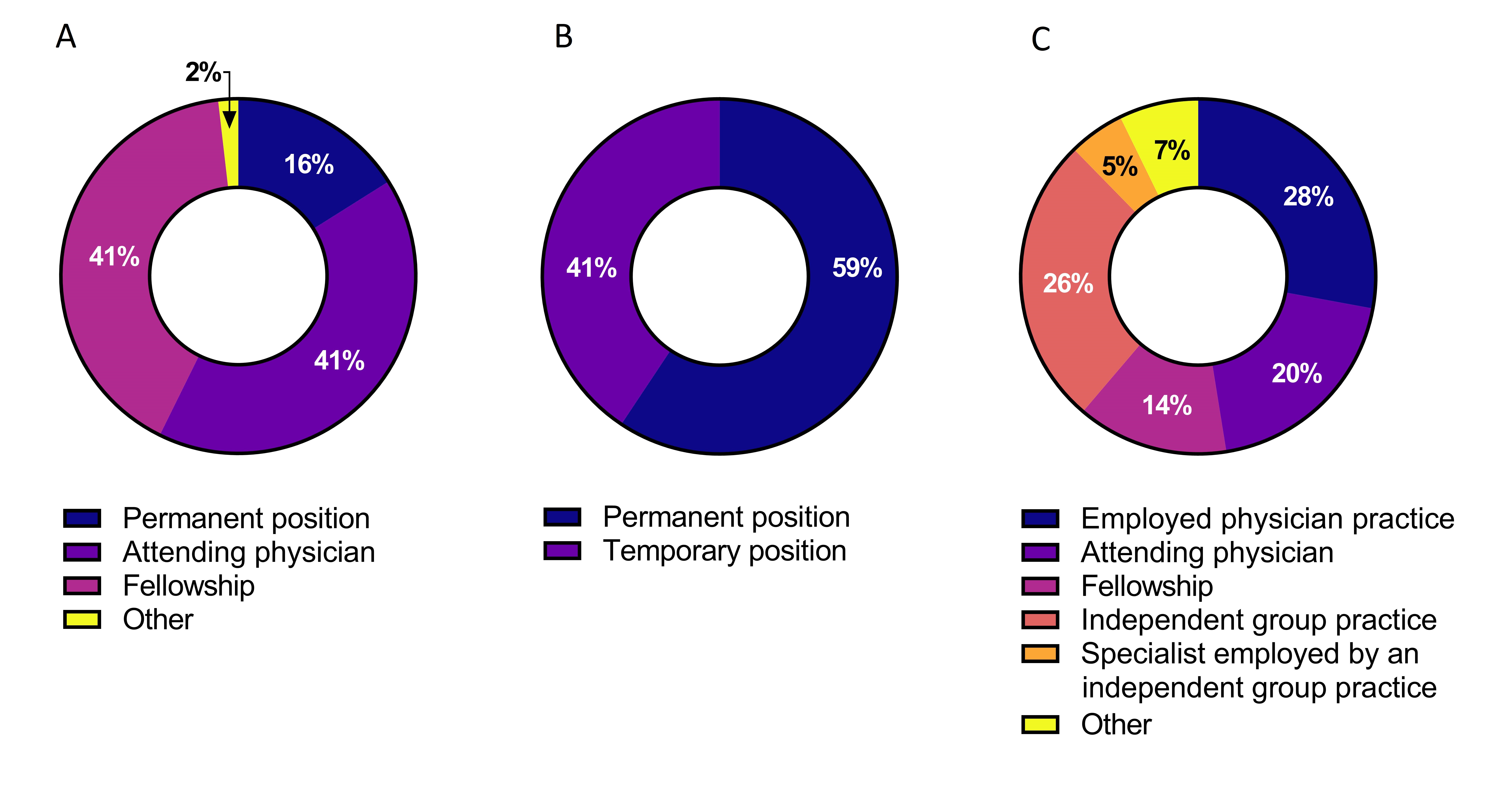

Supplement: Supplementary file 2 — Figure S1 Employment of young cardiologists with regard to a first job, b type of position at time of survey, and c current job, divided into employed physician practice (specialist in dienstverband), attending physician (chef de clinique), fellow, independent group practice (specialist in maatschap), specialist employed by independent group practice (specialist in dienst van maatschap; SPIDMA) and other (e.g. cardiologist in private clinic) [file 12471_2022_1736_MOESM2_ESM.jpg]
